# Emission characteristics and vapour/particulate phase distributions of PCDD/F in a hazardous waste incinerator under transient conditions

**DOI:** 10.1098/rsos.171079

**Published:** 2018-01-10

**Authors:** Min Li, Chao Wang, Kefa Cen, Mingjiang Ni, Xiaodong Li

**Affiliations:** State Key Laboratory of Clean Energy Utilization, Institute for Thermal Power Engineering, Zhejiang University, Hangzhou, Zhejiang Province 310027, People's Republic of China

**Keywords:** PCDD/F, hazardous waste incinerator, transient conditions, gas/particulate phases, PCBs

## Abstract

Polychlorinated dibenzo-p-dioxin and polychlorinated dibenzofuran (PCDD/F) emission characteristics and vapour/particulate phase partitions under three continued operation conditions, i.e. shut-down, start-up and after start-up, were investigated by sampling stack gas. The results indicated that the PCDD/F emission levels were 0.40–18.03 ng I-TEQ Nm^−3^, much higher than the annual monitoring level (0.016 ng I-TEQ Nm^−3^). Additionally, the PCDD/F emission levels in start-up were higher than the other two conditions. Furthermore, the PCDD/F congener profiles differed markedly between shut-down and start-up, and the chlorination degree of PCDD/F increased in shut-down and decreased evidently in start-up. Moreover, PCDD/F vapour/particulate phase distributions varied significantly under three transient conditions. The PCDD/F vapour phase proportion decreased as the shut-down process continued, then increased as the start-up process proceeded, finally more than 98% of the PCDD/F congeners were distributed in the vapour phase after start-up. The correlations between log(*C*_v_/*C_s_*) versus $\text{log }p_{L}^{0}$ of each PCDD/F congener in stack gas were disorganized in shut-down, and trend to a linear distribution after start-up. Besides, polychlorinated biphenyl emissions show behaviour similar to that of PCDD/F, and the lower chlorinated congeners have a stronger relationship with 2,3,7,8-PCDD/Fs, such as M1CB and D2CB.

## 1. Introduction

Waste products generated by the chemical and hospital industries are classified as hazardous waste (HW) in China and include a wide variety of types, compositions and calorific values. Currently, incineration is the ultimate disposal method for HW due to weight and volume reduction, detoxification and energy recovery. However, polychlorinated dibenzo-p-dioxins and polychlorinated dibenzofurans (PCDD/Fs) emitted from hazardous waste incinerators (HWIs) have made HWI an issue of concern because 17 PCDD/F congeners with chlorine substitution in 2,3,7,8 positions are most toxic to humans. Atmospheric transport is the key factor in moving PCDD/Fs from sources to remote regions and in contaminating terrestrial and aquatic environments and the food chain [1]. The atmospheric fate of PCDD/Fs is primarily governed by their vapour/particulate partitioning.

In flue gas, PCDD/Fs are partitioned as gas phase and particle-bound products (particulate phase). To control PCDD/F emission levels and meet the stringent standards, various types of air pollutant control devices (APCDs) would be equipped within HWIs. Vapour phase PCDD/Fs can be removed by various methods including adsorption on carbon-based adsorbents, or catalytic destruction, such as activated carbon injection (ACI) and selective catalytic reduction. While particulate phase PCDDs/Fs are largely controlled by electrostatic precipitator (EP) and bag filters (BFs). Among those devices available for use, ACI to adsorb vapour phase PCDD/Fs and BF to remove particulate matter are considered the simplest. However, the removal efficiencies of PCDD/F congeners achieved with ACI are not always consistent due to the variation of vapour pressure and different adsorbing capacities of the activated carbons for different congeners [2]. Information on PCDD/Fs partitioning in vapour/particulate phases is important in selecting, designing and operating PCDD/F control equipment.

Previous studies found that congener's vapour pressure, particle concentration, flue gas temperature and removal mechanism of the APCDs applied were key parameters in controlling the phase variation. Most of those studies on characteristics of PCDD/F congener distributions in vapour/particulate phases from waste incinerators were under normal operation conditions. Few studies have evaluated the PCDD/F congener partition in vapour/particulate phases under the inevitable transient conditions, such as start-up and shut-down. Lots of researches indicated that PCDD/F emissions from waste incinerators were much higher under transient operation conditions [3–6], especially for HWI. Because the contents of chlorine and heavy metals in HW are generally higher than those in municipal solid waste, which could accelerate the formation of PCDD/F, especially under poor or unstable combustion conditions [6–8]. Moreover, Wang *et al*. [6] found that for a municipal solid waste incinerator (MSWI; consisting of four incinerators), one start-up procedure can generate approximately 60% of the PCDD/F emissions for one whole year of normal operations. And incinerators are usually shut down and started up at least once a year for maintenance, which is unavoidable. Hence, information on which form of PCDD/Fs exists in flue gases under start-up and shut-down processes is important to control PCDD/F emission level during those two conditions.

This research is a series of detailed studies on the changes in emission levels, congener profiles, vapour/particulate phase partitioning under three operation conditions (shut-down, start-up and after start-up). In addition, PCB emissions and the correlation with PCDD/F were studied to obtain more detailed information on the PCDD/F formation mechanism.

## 2. Measurements and materials

**Figure 1.**
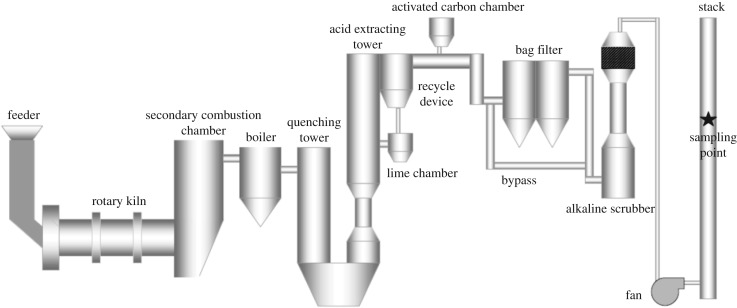
System flow diagram of HWI (24t/d).

The system flow diagram of HWI (24 t/d) investigated in this study is shown in figure 1. The main combustion section consists of a rotary kiln and a secondary combustion chamber. The combustible materials in waste are boiled and oxidized into gases, which are completely burned in the second combustion chamber, and the non-flammable inert solid materials are discharged at the end of the kiln. Generally, the flue gas temperature at the end of the second combustion chamber exceeds 850°C, then decreases quickly after flowing through the boiler and quenching tower. Before discharging to the atmosphere, flue gases must pass through APCDs, including an acid extraction tower, ACI, a BF and an alkaline scrubber (AS).

The waste disposed of in this incinerator is mainly industrial hazardous waste with high thermal value and toxicity, such as waste organic solvents, pesticide waste, medical waste, waste mineral oil, phenol waste, organic phosphides, etc. Owing to the wide range and complex composition, the composition of various types of wastes fluctuates significantly. The main chemical compositions of hazardous waste are C (12–45%), H (1–9%), O (2–13%), S (0.1–0.3%), Cl (0.1–4%), F (0.1–3%), P (0–3%), Si (0–15%), K (0–3%), Na (0–4%), etc. Before being fed into the furnace, hazardous wastes are usually matched together according to their compositions, calorific values and other parameters. Generally, calorific value of the waste was approximately 3500 kcal kg^−1^, chlorine content is below 4%.

### 2.1. Sampling arrangements

**Figure 2.**
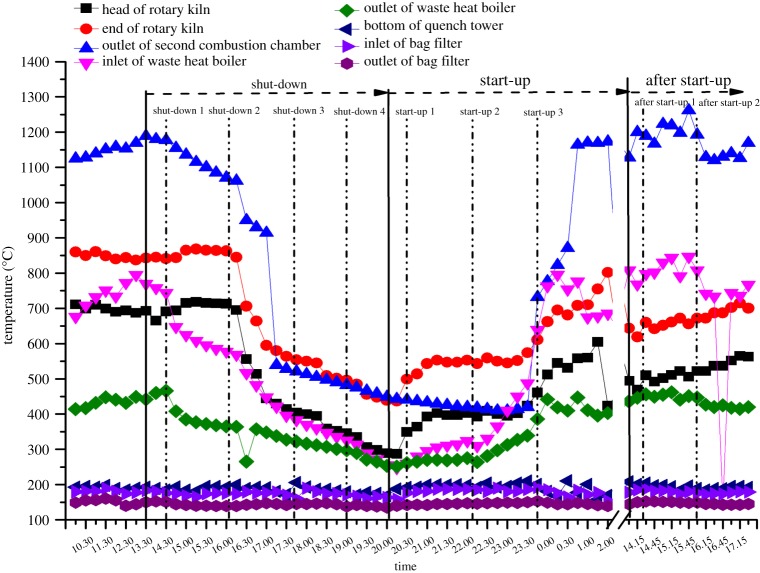
Temperature registration in transient conditions at different points.

In the present study, the flue gas samples were collected at the stack for two days, and the detailed sampling arrangements are shown in table 1. As shown in figure 2, three continued transient operation conditions are depicted in the same figure, temperature versus time was used to describe the whole process. The areas between the solid lines were shut-down, start-up and after start-up conditions, respectively. And dotted lines represented the sampling start time. Before shut-down, the temperature exceeded 850°C at the kiln and was far greater than 1000°C at the second combustion chamber, which was sufficiently high to break up the waste and the organic combustion materials. The sampling in shut-down process began at 14.30 (first day), and the waste feeding and ACI processes were halted simultaneously (table 1) and the first flue gas sample collection began (shut-down 1). As shown in figure 2, the temperature at the kiln and combustion chamber decreased slightly during 1 h after shut-down and subsequently declined markedly, which might have been due to the high induced-draught fan speed used to shorten the shut-down time. At the following sessions, the temperature continued to decrease until 20.30 (first day). Four flue gas samples were collected during shut-down, and the detailed information is recorded in table 1.

**Table 1.** Flue gas sampling and pretreatment arrangements.

	time schedule	designed name	sampling volume (Nm^3^)	flue gas volume (Nm^3^ h^−1^)	ACI	waste feeding	sample	state	pretreatment	pollutants
sample 1	14.30 (first day)	shut-down 1	1.77	19819.23	no	no	gas	particulate	together	PCDD/Fs, PCBs
sample 2	16.05 (first day)	shut-down 2	1.77	21296.43	no	no	gas	particulate	separate	PCDD/Fs
sample 3	17.40 (first day)	shut-down 3	1.82	19098.57	no	no	gas	particulate	together	PCDD/Fs, PCBs
sample 4	19.00 (first day)	shut-down 4	2.00	20614.29	no	no	gas	particulate	separate	PCDD/Fs
sample 5	20.30 (first day)	start-up 1	2.08	17945.71	no	no	gas	particulate	separate	PCDD/Fs
sample 6	22.10 (first day)	start-up 2	2.26	20214.62	yes	yes	gas	particulate	together	PCDD/Fs, PCBs
sample 7	23.45 (first day)	start-up 3	2.19	23042.86	yes	yes	gas	particulate	separate	PCDD/Fs
sample 8	14.22 (second day)	after start-up 1	2.42	19000.23	yes	yes	gas	particulate	separate	PCDD/Fs
sample 9	16.00 (second day)	after start-up 2	2.20	21489.45	yes	yes	gas	particulate	together	PCDD/Fs, PCBs
sample 10	/	blank 1	/	/	/	/	gas	particulate	together	PCDD/Fs, PCBs
sample 11	/	blank 2	/	/	/	/	gas	particulate	separate	PCDD/Fs

The sampling in start-up process began at 20.30 (first day), and temperatures at the kiln and second combustion chamber were less than 500°C at that time. Additionally, flue gas sampling was conducted without waste feeding and ACI. Initially, the temperature at the kiln head increased slowly due to the auxiliary burners operated with diesel oil, and the temperature at the second combustion chamber decreased slightly until waste feeding began (figure 2). As shown in table 1, waste feeding started at 22.30 (first day); subsequently, the temperatures at different points were increased dramatically, especially in the second combustion chamber, where the temperature increased from 500°C to 850°C within 1 h. After waste feeding, two more flue gas samples were collected during start-up. As depicted in figure 2, the temperatures at different points were quite stable after start-up 16 h. Although the temperature at the rotary kiln was not as high as it was before shut-down, the temperature at the second combustion chamber was greater than 1100°C, which was sufficient to break up the residue or incomplete combustion pollutants. Two flue gas samples were collected to investigate the PCDD/F emissions after start-up.

In addition, the contents of CO, oxygen and HCl in the flue gas streams also influence the PCDD/F formation during combustion process. The relevant data for those pollutants in stack gas were noted from the centralized control room.

### 2.2. Sample collection and analysis

Flue gas sampling was conducted using isokinetic samplers (KNJ, Korea) in compliance with US EPA Method 23. The vapour phase sample was collected by absorption on XAD-2 resin, and the particle-bound sample was collected using a fibreglass filter and rinsing the sampling probe thereafter. Owing to the high particle concentration, the fibre filters were replaced three or four times in each flue gas sampling process to make the samples more realistic. A total of nine flue gas samples were collected during the three continued processes. The relevant pretreatment arrangements are depicted in table 1. For a better understanding of PCDD/F vapour/particulate phase distribution under different operation conditions, some of the vapour and particulate samples were treated separately, and the others were treated together. In addition, to investigate the correlation between PCDD/F and PCB in transient conditions, the PCB emissions in the flue gas samples were measured simultaneously.

**Figure 3.**
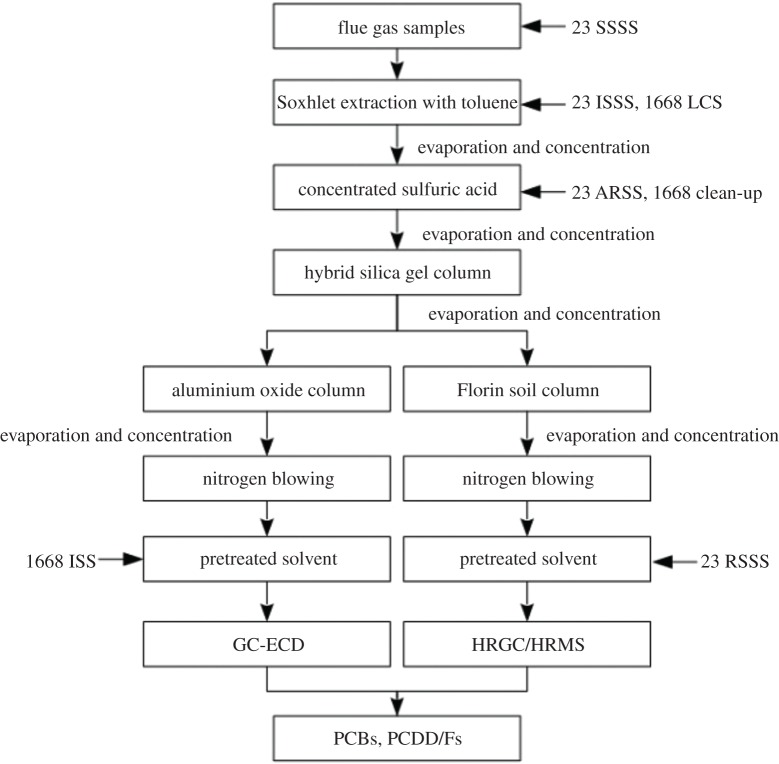
Flow diagram of sample pretreatment.

These flue gas samples were spiked with known amounts of US EPA Method 23 internal standard solution (23 SSSS) prior to sampling to check the PCDD/F sampling efficiency. After sampling, the filters and XAD-2 resin were stored and maintained in dark conditions until transfer to the laboratory. The detailed treatment procedure is depicted in figure 3. The sample was Soxhlet extracted with toluene for 24 h, and the standard solutions 23 ISSS and 1668 LCS for PCDD/F and PCB were added to the samples. After extraction, the toluene extract was concentrated to nearly 1 ml via rotary evaporation and replaced with 5–6 ml of hexane. Subsequently, the hexane solvent was cleaned up with concentrated sulfuric acid. In this process, the clean-up standard solutions were injected, including 23 ARSS and 1668 clean-up for PCDD/F and PCB, respectively. Then the treated solution was enriched to approximately 1 ml and subjected to a hybrid silica gel column (sulfuric acid and sodium hydroxide silica gel). The eluate was concentrated to 1 ml, transferred to a vial, and fixed at a volume of 10 ml. The 10 ml solution was equally divided into two portions (5 ml portion^−1^). One portion was subjected to an aluminium column for PCDD/F detection, and the other was subjected to a Florin soil column for PCB detection. After that, two kinds of eluates were concentrated, transferred to two vials, and subsequently enriched to near dryness via nitrogen blowing. Approximately 30 µl of nonane was injected into the vial and shaken for 2 min in the ultrasonic oscillator. Finally, two kinds of solution were delivered to sample bottles, which had been subjected to the known amount of 23 RSSS and 1668 ISS standard solutions.

Both PCDD/F and PCB were determined with high-resolution gas chromatography (HRGC)/high-resolution mass spectrometry (JMS-800D, JEOL, Japan). The HRGC was equipped with a DB-5MS fused silica capillary column (*L* = 60 m, ID = 0.25 mm, film thickness = 0.25 µm) to separate the PCDD/F and PCB congeners. Helium was used as the carrier gas. Moreover, the temperature-setting program was different for PCDD/F and PCB detection. For PCDD/F, the oven temperature was programmed with an initial temperature of 150°C, increased to 190°C at a rate of 25°C min^−1^, subsequently raised at 3°C min^−1^ to 280°C and held for 20 min. For PCB, the oven temperature was initially set at 75°C, increased to 150°C at a rate of 15°C min^−1^, subsequently raised at 2.5°C min^−1^ to 290°C and held for 1 min. The mass spectrometer was operated with a minimum resolution of 10 000 under positive EI conditions, and the data were obtained in selected ion monitoring (SIM) mode. The electron energy and source temperature were specified at 38 eV and 280°C, respectively.

The QA/QC samples were all analysed concurrently with the collected flue gas samples. The pretreatment arrangements of the blanks are shown in table 1. The measurement results indicated that the PCDD/F levels in the blank samples were all at least three times lower than the detection limits. Moreover, the credibility of the PCDD/F and PCB data was verified by addition of internal standard solutions prior to the Soxhlet extraction, purification and analysis steps, respectively. The recovery rates of each internal standard solution were between 55 and 120%, in accordance with the required recovery standard of 30 to 130%.

## 3. Results and discussion

### 3.1. Polychlorinated dibenzo-p-dioxin and polychlorinated dibenzofuran concentrations

The data of PCDD/F, PCB and other pollutants are shown in table 2. The emission levels of PCDD/F under the three operation conditions were 0.40–18.03 ng I-TEQ Nm^−3^, which were much higher than the annual monitoring level (0.016 ng I-TEQ Nm^−3^).

**Table 2.** Results of PCDD/Fs, PCBs and other pollutants. t, total; g, gas phase; p, particulate phase.

	136 PCDD/F concentrations (ng Nm^−3^)	17 PCDD/F concentrations (ng Nm^−3^)	PCDD/F TEQ (ng I-TEQ Nm^−3^)	PCDF/PCDD ratio	209 PCB concentrations (ng Nm^−3^)	11 PCB concentrations (ng Nm^−3^)	PCB TEQ (pg WHO-TEQ Nm^−3^)	CO (ppm)	HCl (ppm)	O_2_ (%)	particle (mg Nm^−3^)
shut-down 1	31.34^t^	6.36^t^	0.99^t^	3.34^t^	168.06^t^	1.41^t^	19.52^t^	1.5	1.58	15.50	24.33
shut-down 2	25.63^g^	7.82^g^	0.71^g^	4.06^g^	/	/	/	12.06	1.27	16.50	29.56
	6.73^p^	5.95^p^	1.21^p^	5.07^p^							
shut-down 3	12.76^t^	3.28^t^	0.40^t^	2.40^t^	129.91^t^	0.99^t^	7.59^t^	3.83	0.78	17.00	32.00
shut-down 4	42.91^g^	15.96^g^	1.45^g^	2.33^g^	/	/	/	13.50	60.6	17.20	27.68
	25.62^p^	23.73^p^	3.77^p^	5.50^p^							
start-up 1	71.53^g^	5.97^g^	0.63^g^	3.68^g^	/	/	/	62.04	0.49	19.40	33.2
	70.30^p^	6.15^p^	0.64^p^	3.65^p^							
start-up 2	639.02^t^	66.45^t^	7.36^t^	3.06^t^	3116.39^t^	178.75^t^	2055.27^t^	364.13	0.91	16.10	24.29
start-up 3	943.0^g^	48.5^g^	9.65^g^	4.53^g^	/	/	/	1158.20	6.6	14.30	17.51
	132.6^p^	35.36^p^	8.38^p^	5.87^p^							
after start-up 1	83.58^g^	4.96^g^	1.04^g^	5.45^g^	/	/	/	304.1	2.13	12.00	16.57
	0.942^p^	0.194^p^	0.022^p^	3.18^p^							
after start-up 2	134.36^t^	9.65^t^	1.63^t^	3.70^t^	290.17^t^	13.96^t^	301.52^t^	8.92	1.2	11.70	15.59

**Figure 4.**
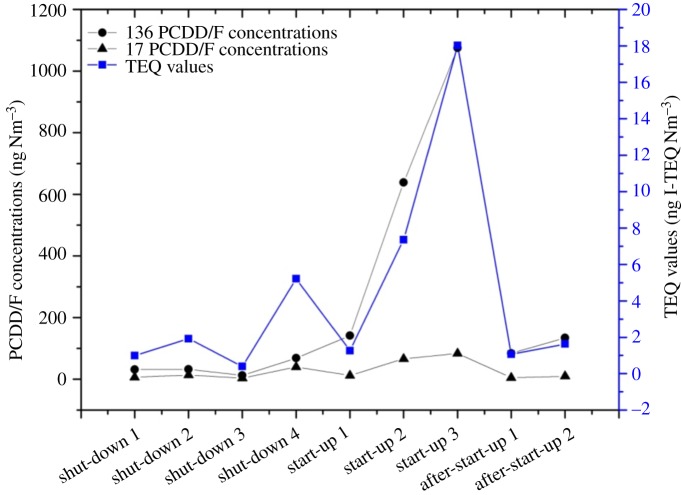
PCDD/F emission levels during three operation conditions.

As depicted in figure 4, the PCDD/F levels at start-up were much higher than those during shut-down and after start-up, similar to previous studies [3,6]. De novo synthesis and precursors are the major pathways of PCDD/F formation in waste incinerators. The former is generated from carbonaceous matter [9] and polycyclic aromatic hydrocarbons at temperatures between 250 and 400°C [10–12]. The latter is produced through condensation reactions of precursors such as polychlorinated biphenyls (PCBs) and chlorophenols at temperatures between 250 and 650°C [13–15]. It is remarkable that the PCDD/F concentration at shut-down 4 increased markedly and was much higher than the other three shut-down stages. Three possible reasons might have caused the rapid growth: (1) waste residues that were burned again during shut-down 4, creating carbon organic pollutants and soot generation, which could accelerate the PCDD/F formation. As shown in table 2, the CO and HCl levels increased visibly in this stage, which confirmed the hypothesis. (2) The temperature at the second combustion chamber outlet ranged from 440 to 470°C in shut-down 4, which covered the optimum temperature of the precursors (250–650°C), much PCDD/F generation possibly occurs in this area. (3) As the flue gas flowed through the cooling section, the temperature at the boiler was near 300°C, which is the optimum temperature for de novo synthesis of PCDD/Fs, thus, large amounts of PCDD/Fs could be generated.

As shown in figure 4, the PCDD/F emission level increased significantly after waste feeding, and the highest level of 18.03 ng I-TEQ Nm^−3^ occurred at start-up 3. The poor and unstable combustion condition resulted in the dramatic elevation. As shown in figure 2, after waste feeding, the temperatures at the rotary kiln and second combustion chamber increased quickly from 550 to 700°C and 420 to 1160°C, respectively. However, most of the temperatures during start-up 2 and 3 were below 800°C, not sufficient for complete breakup. Thus, large amounts of soot, CO (CO content increased to 1158 ppm in start-up 3) and incomplete combustion products (PICs) emerged. Moreover, the formation of fresh soot generated by deficient combustion accelerated the synthesis of PCDD/F because soot freshly formed at high temperature was supposedly highly active [16,17]. As the flue gas flowed, the temperature at the boiler ranged from 250 to 450°C, which is the optimum temperature for PCDD/F synthesis. According to the PCDD/F formation mechanism described previously, a large amount of PCDD/F would possibly be generated at this area.

After 16 h since start-up, the rotary kiln temperature still remained below 800°C, but the second combustion temperature remained stably above 1100°C, which was sufficient to thoroughly burn the combustion gas and residue from the rotary kiln. The average PCDD/F concentration in the stack gas was approximately 1.35 ng I-TEQ Nm^−3^, which is approximately 2.7 times higher than the state limit and 84 times higher than the monitoring level. The high PCDD/F emission after start-up might have resulted from memory effects. According to previous research, two types of PCDD/F memory effect exist, defined by Weber *et al*. [18] as the de novo-based memory effect and the adsorptive memory effect [19]. The de novo-based memory effect might have been caused by high PCDD/F emissions resulting from the start-up procedure or incomplete or disturbed combustion conditions [6,17]. In those situations, the surfaces of the boiler and tube are contaminated by soot particles containing high PCDD/F and hydrocarbons contents, which can improve the de novo synthesis of PCDD/F formation at these locations [17–19]. In addition, the adsorptive memory effect means that PCDD/Fs were desorbed from the filling surfaces of the BFs and scrubbers, which can occur at low temperature and increase the PCDD/F concentrations over a longer period of time. In addition, the adsorptive memory effect is evident when the PCDD/F concentration in flue gas shifts to a lower level. In this study, two memory effects might have resulted in the high PCDD/F level after start-up, but the reasons for the major components are a topic for further study.

### 3.2. Polychlorinated dibenzo-p-dioxin and polychlorinated dibenzofuran congener profiles

**Figure 5.**
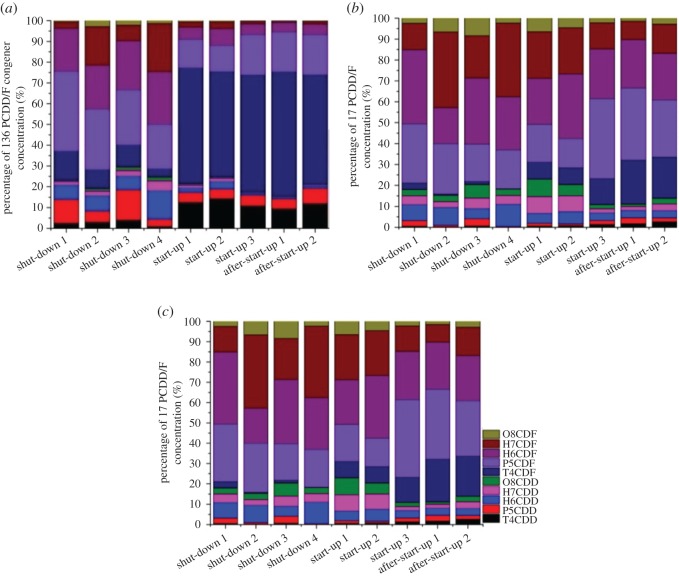
PCDD/F congener profiles during three operation processes. (*a*) 136 PCDD/F concentration, (*b*) 17 PCDD/F concentration and (*c*) 17 PCDD/F TEQ.

The PCDD/F congener profiles are shown in figure 5. It is obvious that PCDFs are the major contributors in the three types of profiles, especially the low-chlorinated PCDF congeners, T4CDF and P5CDF.

**Figure 6.**
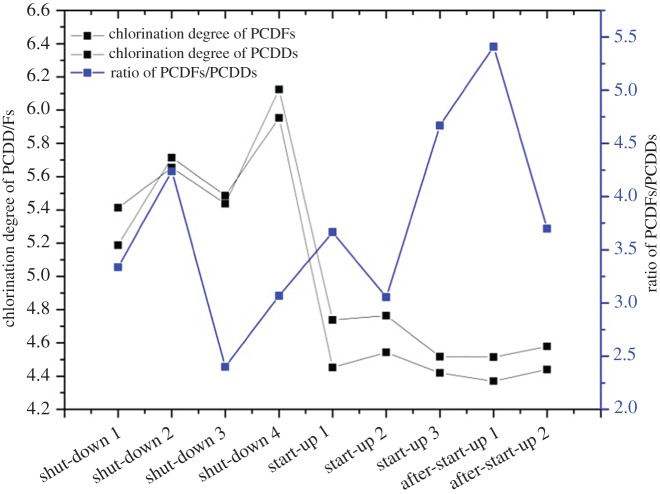
Chlorination degree of PCDD/F and PCDFs/PCDDs ratio.

It is worth noting that the concentration profiles of 136 PCDD/F congeners differed distinctly between shut-down and start-up. P5CDD/F and H6CDD/F were the major contributors in shut-down, whereas T4CDD/F were the main contributors in start-up (figure 5*a*). Additionally, figure 5*a* shows that the proportion of highly chlorinated PCDD/F congeners increased as the shut-down process continued and decreased dramatically as the start-up process proceeded. To clarify this phenomenon, other PCDD/F characteristics were investigated, such as the mass-averaged chlorination degree and PCDFs/PCDDs ratio (P = 4–8) during the three operation conditions, as shown in figure 6. The mass-averaged chlorination degree of PCDD/F increased during shut-down but decreased in start-up. As recorded in table 2, the HCl level in the stack gas at shut-down ranged from 0.78 to 60.6 ppm (average of 16.06 ppm), which is much higher than that at start-up (0.49–6.6 ppm, average of 2.67 ppm). Although no waste feeding occurred in shut-down, carbon residue burning and chlorine accumulated at the deposits on the tube walls and heat exchanger supplied the chlorine sources during shut-down. Thus, the chlorination degree of PCDD/F increased in shut-down because of the high chlorine level. For start-up, the chlorine supplies during start-up 1 were limited, and combustion of diesel oil only produced a low concentration of HCl. However, large amounts of PIC emerged, generating lower chlorinated PCDD/Fs [20]. After waste feeding, the chlorination degree of PCDD/F continued to decrease in start-up 2 and 3, with a greater chlorine source and less diesel oil. The combustion process was not sufficient after waste feeding, and PICs still existed. Moreover, de novo synthesis of PCDD/F was a main pathway after waste feeding because high amounts of PIC (including chlorophenols) were formed and formation of PCDF (especially low-chlorinated) is favoured [15,16,21]. Thus, low-chlorinated PCDF was a major component in start-up. The increase in the PCDFs/PCDDs ratio during start-up was also attributed to de novo synthesis with the ratio of PCDFs/PCDDs ranging from 3.06 to 4.67 due to an increase in the temperature in the combustion chamber and a reduction of incomplete combustion products when the incinerator operated stably.

### 3.3. Vapour/particulate phases distributions of polychlorinated dibenzo-p-dioxin and polychlorinated dibenzofuran

**Figure 7.**
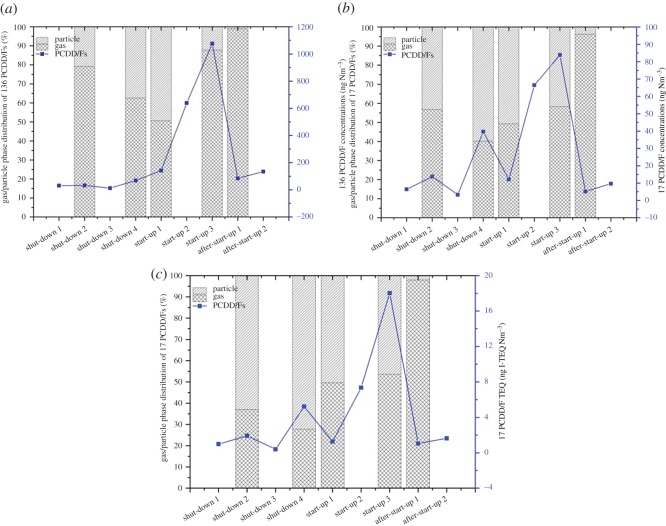
Vapour/particulate phases distributions of PCDD/F. (*a*) 136 PCDD/Fs, (*b*) 17 PCDD/Fs and (*c*) 17 PCDD/F TEQ.

As depicted in figure 7, it is indicated that operation conditions greatly influenced the vapour/particulate phase distribution. The vapour phase proportion of PCDD/Fs decreased rapidly in shut-down and subsequently increased under start-up conditions. After start-up, more than 98% of PCDD/F was distributed in the vapour phase, which agreed with the previous conclusion that the PCDD/F in the stack gas was mostly distributed in the vapour phase under normal operation conditions [1,22].

**Figure 8.**
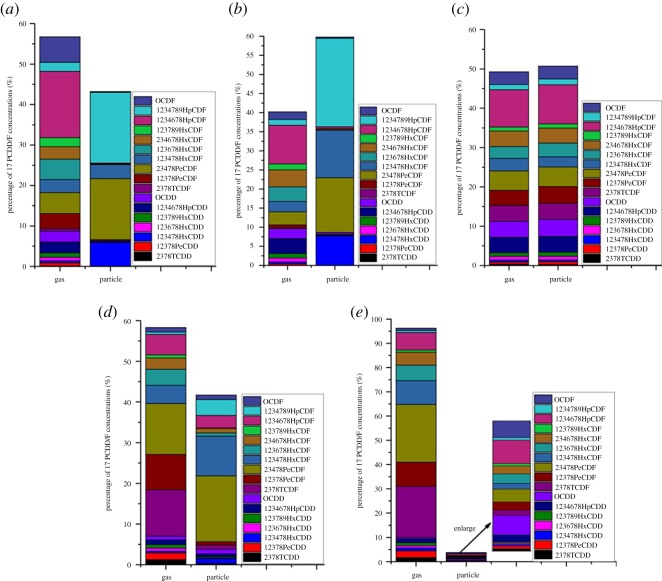
Vapour/particulate phases profiles of 17 PCDD/F congeners in stack gas during different operation conditions. (*a*) Shut-down 2, (*b*) shut-down 4, (*c*) start-up 1, (*d*) start-up 3 and (*e*) after start-up 1.

As shown in figure 7, the vapour/particulate phase distributions of PCDD/F in flue gas were quite different under different operation conditions. All of the PCDD/F TEQ proportions in the particulate phase, except after start-up 1 stage, were higher than those in the PCDD/F concentration. For instance, the TEQ proportion in the particulate phase at shut-down 2 was 63.02%, whereas the relevant concentration proportions of 136 and 17 PCDD/F were 43.02% and 20.08%, respectively. These observations contradict the results of previous studies, in which the PCDD/F TEQ proportions in the particulate phase were lower than those in the PCDD/F concentration [1]. To clarify the contradiction, the vapour/particulate phase profiles of 17 PCDD/F congeners in stack gas under different operation conditions are depicted in figure 8. Figure 8 shows that 2,3,4,7,8-PeCDF, 1,2,3,4,7,8,9-HpCDF, 1,2,3,4,6,7,8-HpCDF and 1,2,3,4,7,8-HxCDD were the major contributors in the particulate phase. The 2,3,4,7,8-PeCDF proportions in the particulate phase were higher than those in the vapour phase. In addition, the relevant toxic equivalent factor (TEF) of 2,3,4,7,8-PeCDF (TEF = 0.5) was higher than the other congeners, such as HpCDF (TEF = 0.01), HxCDF (TEF = 0.1) and OCDD/F (TEF = 0.001). Both reasons explain why the TEQ proportions in the particulate phase were higher than those in the PCDD/F concentration. However, 2,3,4,7,8-PeCDF at after start-up 1 was primarily distributed in the vapour phase, and OCDD and OCDF (TEF = 0.001) were the major contributors in the particulate phase; thus, the particulate phase proportion of PCDD/F TEQ was lower than that in the vapour phase, similar to previous findings which were investigated under normal operation conditions [1,22].

To compare the partitioning of seventeen 2,3,7,8-substituted PCDD/F congeners between vapour and particulate phases in flue gas under transient conditions, data collected are further analysed. The vapour/particulate distribution coefficient for each congener was calculated from the equation (3.1):

3.1 $$\varphi =\log\;\left(\frac{C_{v}}{C_{s}}\right),$$

where *C*_v_ is the concentration of PCDD/F congener existing in vapour phase (ng Nm^−3^) and *C*_s_ is the concentration of PCDD/F congener adsorbed on particles (ng Nm^−3^). *φ* is used to represent the coefficient of vapour/particulate phase PCDD/F congeners partition in logarithm. When the *φ* value is greater than 0, it indicates that over 50% PCDD/F congeners are distributed in vapour phase. Previous study indicates that vaporization is the major mechanism that causes particulate phase PCDD/Fs to transfer into vapour phase (especially for the lower chlorinated congeners, like TCDD) [23]. Hence, the coefficient of semivolatile compounds (like PCDD/Fs) adsorbed to particles was mainly affected by the vapour pressures of those compounds. Furthermore, the vapour pressures of PCDD/F congeners increase as the gas temperature increases. Previous studies found that saturation vapour pressure $\left(p_{L}^{0}\right)$ values for the PCDD/Fs were correlated with GC-RI values on a DB-5 GC column, nCl, molecular weight and melting point [24,25]. Mader & Pankow [26] developed a consistent set of parameters for estimating the temperature-dependent $p_{L}^{0}$ values for all 210 PCDD/Fs with the well-known Kirchoff equation (3.2).

3.2 $$\log\;p_{L}^{0}(T)=A-\frac{B}{T}+C\log\;T,$$

where $p_{L}^{0}$ is the saturation vapour pressure of the organic compound and *T* is the temperature (K). The related parameters *A*, *B*, *C* for 2,3,7,8-substutited PCDD/F congeners are shown in table 3.

**Table 3.** Parameters for the calculation of $p_{L}^{0}$ for 2,3,7,8-substutited PCDD/F congeners as a function of temperature [26].

	*A*	*B*	*C*
2378-TCDD	46.89	7061	−11.499
12378-PeCDD	44.87	7079	−10.971
123478-HxCDD	42.99	7132	−10.437
123678-HxCDD	42.96	7132	−10.437
123789-HxCDD	42.9	7130	−10.437
1234678-HpCDD	40.88	7142	−9.904
OCDD	38.26	6985	−9.37
2378-TCDF	35.91	6019	−8.399
12378-PeCDF	34.15	6081	−7.865
23478-PeCDF	33.99	6084	−7.865
123478-HxCDF	32.14	6106	−7.332
123678-HxCDF	32.12	6108	−7.332
234678-HxCDF	31.99	6108	−7.332
123789-HxCDF	31.92	6114	−7.332
1234678-HpCDF	30.03	6090	−6.798
1234789-HpCDF	29.69	6089	−6.798
OCDF	27.96	6148	−6.267

**Figure 9.**
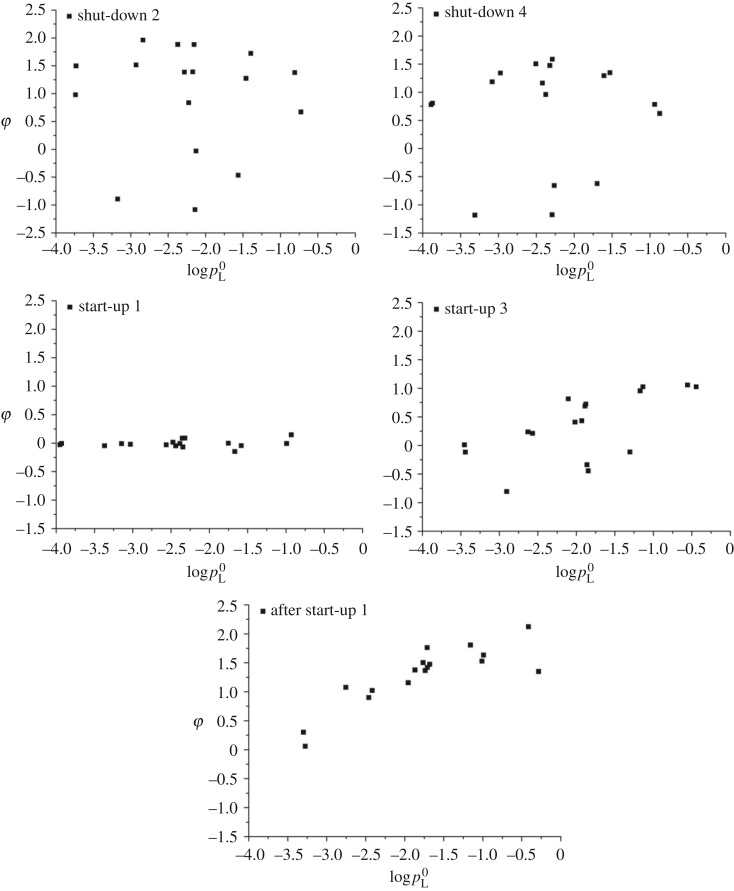
The coefficient of vapour/particulate phase PCDD/F partitioning in stack gas.

Figure 9 shows the trends between the log(*C*_v_/*C*_s_) versus $\log\;p_{L}^{0}$ of each PCDD/F congener in stack gas at different operation conditions. Each dot represents the log(*C*_v_/*C*_s_) versus $\log\;p_{L}^{0}$ of each seventeen 2,3,7,8-substituted PCDD/F congener in flue gas. As depicted in figure 9, the dot distributions at different conditions are very different, the dot distributions in shut-down are messy, while the distributions in start-up are more organized and trend to be linear, especially after start-up. Two possible reasons caused those phenomena, one was no ACI in shut-down 2, shut-down 4 and start-up 1. According to previous studies, the PCDD/F log(*C*_v_/*C*_s_) versus $\log\;p_{L}^{0}$ at the cyclone outlet under normal operation condition in MWI were still in good linear correlation. Thus, the ACI has little impact on those disorganized dot distributions. The other reason was the unstable operation conditions, and the detailed information was needed to be further studied. Moreover, the values of log(*C*_v_/*C*_s_) in shut-down are mostly greater than 0, except for 1,2,3,4,7,8-HxCDD, 2,3,4,7,8-PeCDF, 1,2,3,4,7,8-HxCDF and 1,2,3,4,7,8,9-HpCDF. However, lots of the values log(*C*_v_/*C*_s_) in start-up are less than 0, those values are much close to 0, which means the PCDD/F vapour/particulate phase partition were nearly to 50%. After start-up, the log(*C*_v_/*C*_s_) values are all greater than 0, the operation conditions were close to stable and normal, and most of the PCDD/Fs were distributed in vapour phase, which was similar to previous studies [2].

### 3.4. Correlation of polychlorinated dibenzo-p-dioxin and polychlorinated dibenzofuran with polychlorinated biphenyl

**Figure 10.**
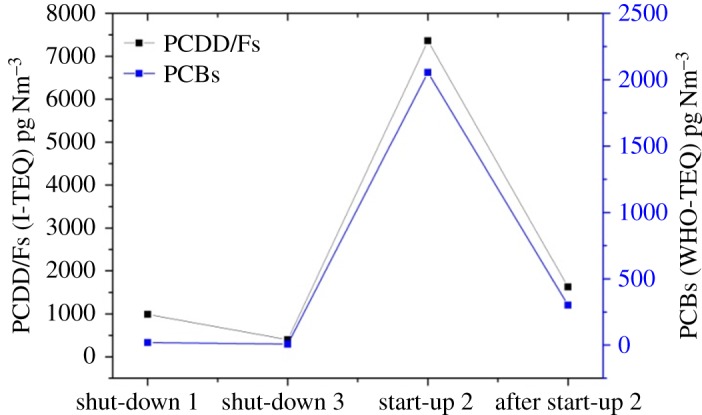
TEQ correlation between PCDD/F with PCB.

**Figure 11.**
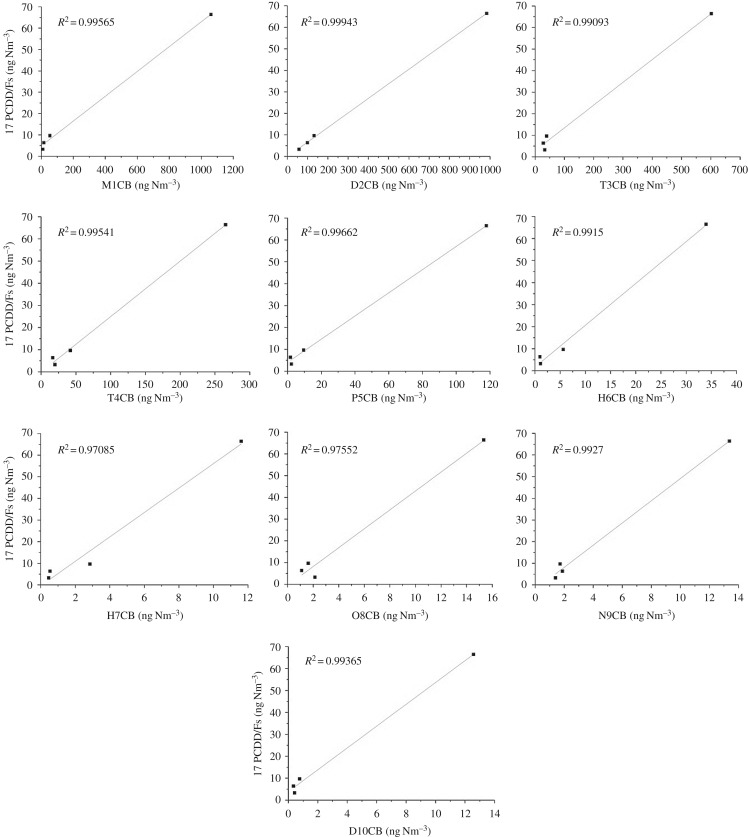
Correlation between 209 PCB congeners with 2,3,7,8-PCDD/Fs.

As shown in figure 10, the trends of PCB emission during the three operation conditions were similar to those of PCDD/F. The highest PCB emission was similar to the behaviour of PCDD/F in start-up 2. The similarity between the PCB emission levels with PCDD/F might have been due to the observation that PCB serves as precursors, i.e. reactants, to form PCDD/F. Because of the toxicity of 2,3,7,8-PCDD/F, the correlation between the total PCBs (209) with 2,3,7,8-PCDD/Fs was investigated (figure 11). Regression analysis was used to investigate the concentration correlation between PCDD/Fs with PCB congeners, as shown in figure 11. *R*^2^ is the coefficient of determination, which ranges from 0 to 1. And the closer the value of *R*^2^ is to 1, the stronger the association between PCDD/F with PCB congener. Most of the *R*^2^ values were above 0.99 in figure 11, very close to 1, which means the correlation seems good between PCDD/F and PCB congeners. And less-chlorinated PCB congeners such as M1CB, D2CB were more strongly correlated with 2,3,7,8-PCDD/F concentrations. As shown is figure 11, a point stayed far away from the other three points, which belonged to start-up 2. Because the PCDD/F and PCB concentrations in start-up 2 were higher than those in shut-down 1, shut-down 3 and after start-up 2 processes.

## 4. Conclusion

The aim of this study was to investigate the PCDD/F emission characteristics of a HWI under three common transient operation conditions. The results indicated that PCDD/F concentrations generated during transient conditions, were much higher than those under normal operation conditions. Temperatures at the end of the second combustion chamber ranged from 420 to 540°C, which would cause large amounts of PCDD/F to be generated in this area and led to a higher emission level. Thus, during the shut-down and start-up processes, the temperature at the second combustion chamber should be quickly decreased or increased to avoid this temperature region. Moreover, the PCDD/F congener profiles indicated that PCDFs were the major contributors to PCDD/F concentration and TEQ. P5CDF and H6CDF were the major contributors to PCDD/F emission in shut-down, whereas T4CDF was the main part in start-up. The vapour/particulate phase distributions of PCDD/F were influenced significantly by transient operation conditions. The PCDD/F TEQ proportions in the particulate phase were higher than those in the concentrations under shut-down and start-up conditions. The PCB emissions behaviour was similar to that of PCDD/F, and M1CB and D2CB were the congeners most strongly correlated with 2,3,7,8-PCDD/F concentration. In a word, PCDD/Fs and PCBs are much easier to generate under transient conditions, causing the fluctuated temperature and unstable combustion provided a lot of favourable conditions, so operators should pay more attention to those processes.

## Data accessibility

This article has no additional data.
